# Ecological and Genetic Divergences with Gene Flow of Two Sister Species (*Leucomeris decora* and *Nouelia insignis*) Driving by Climatic Transition in Southwest China

**DOI:** 10.3389/fpls.2018.00031

**Published:** 2018-01-25

**Authors:** Yujuan Zhao, Genshen Yin, Yuezhi Pan, Xun Gong

**Affiliations:** ^1^Key Laboratory for Plant Diversity and Biogeography of East Asia, Kunming Institute of Botany, Chinese Academy of Sciences, Kunming, China; ^2^Department of Biological Science and Technology, Kunming University, Kunming, China

**Keywords:** climatic shift, coalescent analyses, endemic species, gene flow, niche differentiation, speciation

## Abstract

Understanding of the processes of divergence and speciation is a major task for biodiversity researches and may offer clearer insight into mechanisms generating biological diversity. Here, we employ an integrative approach to explore genetic and ecological differentiation of *Leucomeris decora* and *Nouelia insignis* distributed allopatrically along the two sides of the biogeographic boundary ‘Tanaka Line’ in Southwest China. We addressed these questions using ten low-copy nuclear genes and nine plastid DNA regions sequenced among individuals sampled from 28 populations across their geographic ranges in China. Phylogenetic, coalescent-based population genetic analyses, approximate Bayesian computation (ABC) framework and ecological niche models (ENMs) were conducted. We identified a closer phylogenetic relationship in maternal lineage of *L. decora* with *N. insignis* than that between *L*. *decora* and congeneric *Leucomeris spectabilis*. A deep divergence between the two species was observed and occurred at the boundary between later Pliocene and early Pleistocene. However, the evidence of significant chloroplast DNA gene flow was also detected between the marginal populations of *L. decora* and *N. insignis*. Niche models and statistical analyses showed significant ecological differentiation, and two nuclear loci among the ten nuclear genes may be under divergent selection. These integrative results imply that the role of climatic shift from Pliocene to Pleistocene may be the prominent factor for the divergence of *L*. *decora* and *N*. *insignis*, and population expansion after divergence may have given rise to chloroplast DNA introgression. The divergence was maintained by differential selection despite in the face of gene flow.

## Introduction

Studying the driving forces which promote species diversification is a major interest in diversity and evolutionary research (Mayr, 1942). Species divergences have often been driven by geographic isolation caused by geological events or major climatic fluctuations, resulting in genetic change or even divergent ecological traits (Mayr, 1947). The geographic isolation is generally considered as the prerequisite for the geographical mode of predominantly allopatric speciation, where gene flow among splitting populations is completely disrupted by physical barriers, and genetic divergence arises via local adaptation, mutation and genetic drift (Coyne and Orr, 2004). However, the accumulation of recent studies assessing gene flow between diverging species, suggests that divergence with gene flow is an important driver for generating biological diversity (e.g., Li et al., 2014; Wang et al., 2016). This raises the question how population become genetically isolated despite the homogenization effect of gene flow and what factors facilitate subsequent speciation (Feder et al., 2012).

Species divergence engendered by ecological process (niche shift, expansion or specialization), in which adaptation to different ecological niches can create productive barriers between species through ecologically based divergent selection (Rundle and Nosil, 2005), has been recently demonstrated by empirical studies (Nosil et al., 2009; Li et al., 2014; Oneal et al., 2014; Gould et al., 2017). Comparatively, under ecological selection, migration may occur between populations that are in close proximity but adapted to distinct niches. However, the potential for genetic exchange could be limited, because the fitness of immigrants of hybrids may be less than that of an existing population in a given environment (Nosil et al., 2005). Thus, during the process of species divergence under the circumstance of divergent selection, genetic divergence may be still maintained even if without the physical barriers, and ecological differentiation reflects a balance between natural selection and the homogenization effect of gene flow (Abbott et al., 2008; Feder et al., 2012; Anacker and Strauss, 2014).

Actually, it is not easy to distinguish between strictly allopatric isolation mode and sympatric speciation, because local adaptation could play a role in both scenarios, since the diverging lineages after allopatric speciation could have adapted to different climates and limit gene flow when coming back together (Feder et al., 2013). To determine the relative contributions of geography and ecological factors promoting species diversification, it requires the combination of analyses of possibly historical geographical separation of taxa based on informative molecular markers and environmental conditions that shape the current distribution and ecological requirements of species (e.g., Zhou et al., 2012; Marske et al., 2013; Ortego et al., 2015).

The mountains of Southwest China has a complex geographic history, with deep topographic and climatic gradients that shaped one of the world’s major centers of plant and animals diversity, offering a natural laboratory for studying lineage diversification and speciation in a geographical context (Wu et al., 2005; Lopez-Pujol et al., 2011). Here we present the evolutionary history of two closely related plant species distributed across the ‘Tanaka Line’ in Southwest China (Li and Li, 1992, 1997). The two species concerned, belonging to two genus *Nouelia* and *Leucomeris*, which are sister groups within the tribe Mutisieae (Asteraceae) (Anderberg et al., 2007) and recently were placed within Hyalideae (Panero and Funk, 2008; Funk et al., 2014). *Nouelia* is a monotypic genus and *Leucomeris* is currently divided into two species: *L*. *decora* and *L*. *spectabilis* in Nepal (Panero and Funk, 2008). The morphological characters of *N. insignis* are similar to those of *L*. *decora* with the exception of the inflorescence feature (see Zhao and Gong, 2015). Previous analyses supported a sister relationship between *L*. *decora* and *N. insignis* (Lin, 2007), however, the interspecific relationships among the three species have not been determined.

These two species show almost allopatric distribution on each side of the ‘Tanaka Line’ (except for ES population) and different habitat preferences. The woody *L*. *decora* is widely distributed in valleys or at the edge of the forest and isolated mountaintops in Southwest Yunnan China. *L*. *decora* also has Myanmar, Thailand, and Vietnam records, and generally associated with a warmer and wetter climate due to their occurrence at lower latitudes and altitudes (average 1550 m). In contrast, the woody *N*. *insignis* is endemic and confined to a small area along the dry-hot valleys in Yunnan and Sichuan China with an average altitude of 1800 m, which is featured by aridity even in the rainy season (Jin, 1999). Our previous analyses have revealed that the two species share common haplotypes in their chloroplast DNA, but the level of interspecific divergence of nuclear DNA is much higher without the presence of strong gene flow detected (Zhao and Gong, 2015). Therefore, given the geographic distribution of *N*. *insignis* in a geologically and climatically dynamic area and the close relationship with *L*. *decora* observed in preliminary analyses of cpDNA (Lin, 2007; Panero and Funk, 2008), these species provide a good system for test hypotheses related to geographic or climatic modes of lineage divergence using multi-locus sequence data.

In this study, we used an integrative approach to test the alternative hypotheses for the divergence of *N*. *insignis* and *L*. *decora* driving by possible roles of geological or climate events. Firstly, we tested our hypothesis by further examining whether the endemic *N*. *insignis* is closer to *L*. *decora* inferred from both multi-locus of cpDNA and nDNA data sets, based on the phylogenetic analysis of Panero and Funk (2008). Although a previous study suggested a sister relationship between these two species, the samplings only contained species within eastern Asian Mutisieae and other relative species such as *L*. *spectabilis* was not included. Therefore, evaluating the phylogenetic relationships among the three species would be worthwhile. Secondly, given that *N*. *insignis* and *L*. *decora* were indeed identified as sister taxa in our study, we next quantified the level of gene flow as well as other demographic parameters during the species formation between *N*. *insignis* and *L*. *decora* to reveal the possible geographic mode of speciation. We analyzed patterns of interspecific genetic differentiation and employed an approximate Bayesian computation (ABC) framework to test the hypothesis for the divergence of *N*. *insignis* and *L*. *decora* and explore which possible mechanisms (geological or climate events) might have caused differentiation of the two species under the different scenarios. Third, using the ecological niche modeling, the ecological requirements of the two species in their distribution ranges were compared and changes in species distribution as well as tests of niche divergence were also modeled. These integrative analyses can help infer population demographic history and understand it in the light of past environmental changes. Together, examining these questions not only provides insights into the evolutionary history of closely related species, but also contributes to understanding the high endemism in southwest China.

## Materials and Methods

### Plant Materials

We sampled 12 populations of *L*. *decora* and 16 of *N*. *insignis*, respectively (Supplementary Table S1). Additionally, samples of *Pertya phylicoides* and *Ainsliaea latifolia* were also collected to serve as outgroups for nuclear phylogenetic analyses, for we failed to obtain the sampling of *L*. *spectabilis* in Nepal and these two genus were shown to be closely related with *Leucomeris* and *Nouelia* within the Mutisieae (Asteraceae) (Lin, 2007).

### Sequencing cpDNA and Phylogenetic Analyses

To assess the phylogenetic relationship of *L*. *decora* and *N*. *insignis*, nine chloroplast genes were sequenced for three individuals per population using previously designed primers (*matK* including *trnK*, *ndhD*, *ndhF*, *ndhI*, *rbcL*, *rpoB*, *rpoC*, *trnL*-*trnF*, and *23S*-*trnA*) (Panero and Funk, 2008). All PCR products were directly sequenced from both directions and c. 10,616 bp from nine loci were obtained. The newly obtained sequences were deposited in the GenBank (KX438061-KX438095). Eighteen haplotypes were identified from 81 individuals and then aligned with those retrieved from GenBank for other Wunderlichiodeae species (Supplementary Table S2) (Panero and Funk, 2008) using Clustal X. In total, 25 sequences were included.

A maximum-likelihood (ML) tree was estimated with RAxML version 8.0 (Stamatakis, 2006) The GTRGAMMA model was applied to all genes as the general time reversible (GTR) is the only substitution model implemented in RAXML. A bootstrapping procedure with 1000 replicates was performed to evaluate the significance of branches.

### Phylogeographic Analyses of CpDNA

To further detect the phylogeographic pattern of cpDNA sequences, two more chloroplast fragments (*rpl16* and *trnL*-*rpl32*) were added for analyses after the complementary sequencing of increasing populations. A total of 261 individuals across 28 populations of *L*. *decora* and *N*. *insignis* were used for amplification and sequencing following the methods described by Gong et al. (2011) and Zhao and Gong (2015). The cpDNA haplotype network was constructed using NETWORK v. 5.0 to examine genealogical relationships among haplotypes^1^.

### Sequencing of Multiple Nuclear Genes and Species Delimitation

Ten nuclear genes were successfully sequenced for 5 individuals each population after preliminary screening from single or low copy nuclear genes. Polymerase chain reaction amplification of seven loci (*A27*, *A39*, *B27*, *C12*, *C44*, *D22*, *D34*), which were developed of ESTs from lettuce and sunflower and genomic sequences of *Arabidopsis* and characterized as Conserved Ortholog Set (COS) of low or single-copy genes, were conducted following the methods described by Chapman et al. (2007) (Supplementary Table S3). The amplification of two loci (*AroB* and *GA2ox1*) were attempted with previously reported primers (Li et al., 2008; Mitsui and Setoguchi, 2012) and one locus (*GAPDH*) using primers designed for our previous study (Zhao and Gong, 2012) (Supplementary Table S3). Haplotype phasing was carried out by applying the PHASE algorithms in the software package DnaSP v. 5.0 (Librado and Rozas, 2009). If phase probabilities of nucleotide sites (other than those containing singletons) were below 0.85 after computation (Harrigan et al., 2008), we resolved the gametic phases of selected individuals by cloning. The obtained haplotypes were deposited in GenBank under accession numbers KX438096-KX438315. A species tree was estimated from the joint posterior probability of the nuclear gene trees using software BEAST v. 1.8.2 (Drummond and Rambaut, 2007). We analyzed the dataset including all individuals used for nuclear DNA sequencing. For details on the analysis settings, see Supplementary Note S1.

### Genetic Diversity and Interspecific Divergence

For each nuclear locus, we calculated the basic population genetic parameters, including the number of segregating sites (S), Watterson’ s parameter (θ_w_), nucleotide diversity (π), the minimum number of recombinant events (Rm) and number of haplotypes (N_h_) in the software package DnaSP v. 5.0 (Librado and Rozas, 2009). To quantify the extent of population genetic structure, genetic divergence per nuclear locus between species measured in terms of the fixation index F_ST_ was computed in ARLEQUIN v. 3.1.1 (Excoffier et al., 2005). The significance was determined by the test of 10,000 permutations. Besides, four different methods were employed to detect genetic grouping of all individuals sequenced for nuclear genes, including Neighbor-Net (N-Net) uncorrected distance and EqualAngle splits transformation method in SPLITSTREE v. 4.14.4 (Huson and Bryant, 2006), neighbor-joining (NJ) in MEGA v. 7.0, principle component analysis (PCoA) based on a pairwise Euclidian distance matrix using GenAlEX 6.1 (Peakall and Smouse, 2006) and Bayesian clustering of multilocus genotypes using STRUCTURE v. 2.3.4 (Pritchard et al., 2000). The significance of each node was evaluated by bootstrapping with 1000 replications in NJ analyses. We ran STRUCTURE using the admixture model with correlated allele frequencies to account for possible ancestral admixture. The run used a burn-in period of 50,000 Markov chain Monte Carlo (MCMC) generations followed by 500,000 interactions for *K* = 1 through 10 with 20 replicates for each K. We performed the ΔK method (Evanno et al., 2005) to select the best-supported number of clusters using STRUCTURE HARVESTER. We then used CLUMPP v. 1.1.2 (Jakobsson and Rosenberg, 2007) to average each individual’s admixture proportions over the 20 replicates for the best K, and the graphical display results was produced in DISTRUCT v. 1.1 (Rosenberg, 2004).

### Ecological Niche Modeling

The geographic distributions of suitable habitats for each species in the present day, and during Last Glacial Maximum (LGM; 21 ka) were predicted using MAXENT 3.3 (Phillips et al., 2006). The environmental layers comprising 19 bioclimate variables and altitude data package were downloaded from the WORLDCLIM database with resolution 2.5 arc-minute resolution for the present and the LGM. The LGM climate layers are based on the community climate system model (CCSM) and the distribution model was also projected to the LGM under the Model for Interdisciplinary Research on Climate (MIROC) (Hasumi and Emori, 2004). In order to minimize biased fitting of the niche models, the pairwise correlation among the 20 variables was assessed using ENMTOOLS (Warren et al., 2010). Those pairs with correlation coefficients of *r* > 0.75 were discarded (Nakazato et al., 2010; Thode et al., 2014). Accordingly, a final set of eight environmental variables, including altitude and seven bioclimatic variables were selected to construct the models. Species occurrence data were obtained from sampling points as well as from herbarium records available at the Chinese Virtual Herbarium (CVH^2^), and duplicated points were eliminated from the database. We include a total of 49 reliable point localities subjected to the ENMs. Model evaluation statistics were produced from ten cross-validation replicate model runs with 20% of the data used for model testing and overall model performance was evaluated using the area under the curve (AUC) of the receiver operating characteristics (ROC).

To compare the degree of niche overlap between ENMs for the two-species models, niche comparisons were carried out in ENMTOOLS (Warren et al., 2010). Two test statistics were calculated: Schoener’s *D* and standardized Hellinger distance (calculated as *I*) (Schoener, 1968; Warren et al., 2008). We first calculated the actual niche overlap between the two species. Next, we conducted a niche identity test to compare the overlap of the species pair’s actual niches to a distribution of niche overlap obtained from pseudoniches (*n* = 200 pseudoreplicates) generated from a random sampling from the data points pooled for the pair of species. We also used background test to determine whether the species’ niches were more or less distinct than expected based on the environmental background differences between the two taxa. For both directions in the background test, 200 replications were used to calculate the null distribution. The observed values of *D* and *I* between species were compared to the null distribution of *D* and *I* values and the significance was tested using SPSS version 16.0.

### Coalescent-Based Analyses

Isolation with migration analyses were implemented in IMa (Hey and Nielsen, 2007). We investigated interspecific gene flow using the two cpDNA fragments and eight nuclear loci after excluding two loci (*A39* and *GA2ox1*), which deviated from the neutral model with the aid of MFDM neutral test (see Supplementary Note S1), respectively. In addition, the statistic of Tajima’s *D* was tested for the neutrality of the examined loci using ARLEQUIN v. 3.1.1 (Excoffier et al., 2005). The longest non-recombining regions for each locus obtained from IMgc analyses (see Supplementary Note S1) were used for IMa estimation.

We used DIYABC v. 2.1.0 for the ABC-based scenario comparisons (Cornuet et al., 2014) based on the sequence data of the 10 nuclear loci. According to the much lower genetic diversity in *N*. *insignis* than those of *L*. *decora*, together with the phylogenetic analyses (see results), two possible scenarios for divergence of *L*. *decora* and *N*. *insignis* were compared: (1) *L*. *decora* and *N*. *insignis* diverged from the common ancestor and *N*. *insignis* experienced bottlenecks during the glacial period; (2) *N*. *insignis* was derived from *L*. *decora* populations and established its current distribution through recent expansion (Supplementary Figure S1 and Supplementary Table S4). The detailed setting for this analysis was illustrated in Supplementary Note S1.

## Results

### Phylogeny and Phylogeography of CpDNA

**FIGURE 1:**
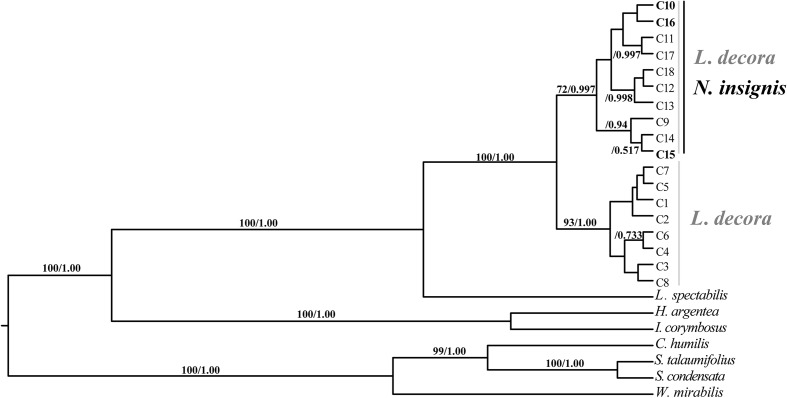
BEAST-derived chronograms of cpDNA haplotypes as well as other Wunderlichiodeae species. Posterior probability and bootstrap values (>50%) based on maximum likelihood (ML) analysis are labeled above branches.

**FIGURE 2:**
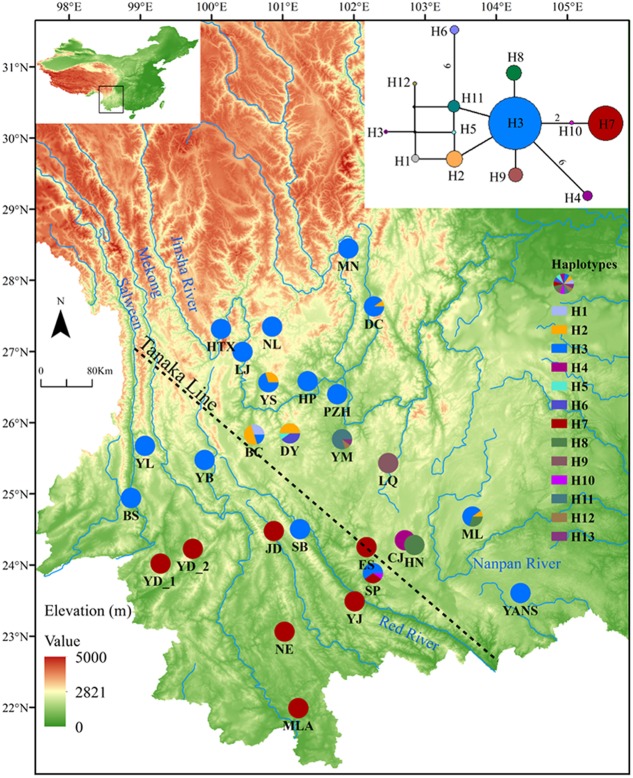
Geographical distribution and network of haplotypes for cpDNA of *L*. *decora* and *N*. *insignis*. Numbers on branches indicate the number of mutations when branches represent more than one mutation. The dash line shows the location of ‘Tanaka Line.’

Sequences of nine cpDNA fragments (in total c. 10,616 bp) were obtained from 81 individuals of *L*. *decora* and *N*. *insignis*, and together, 18 haplotypes were determined, of which three haplotypes (C10, C15, C16) were specific to *N*. *insignis* and the rest to *L*. *decora*. The ML tree of these 18 haplotypes plus those retrieved from GenBank for other Wunderlichiodeae species (Panero and Funk, 2008) showed that these haplotypes formed a well-supported monophyletic group and then clustered with *L*. *spectabilis* (bootstrap, 100%) (**Figure 1**). This result was in agreement with the previous paper, in which *Leucomeris* and *Nouelia* were sister group (Panero and Funk, 2008). However, all the haplotypes of *N*. *insignis* were nested with one clade of *L*. *decora* and did not form a monophyletic clade. The combined analysis of the two cpDNA regions (*rpl16* and *trnL*-*rpl32*), with a total length of 1783 bp, yielded 13 haplotypes. Of those, two were specific to *L*. *decora* (H7 and H10) and others were private for *N*. *insignis*, whereas H3 was shared between them (**Figure 2**).

### Variation in Nuclear Sequences

A total of 137 individuals were sequenced at 10 nuclear loci and yielded a concatenated length of 6584 bp. The number of haplotypes varied from 1 to 24, and comparatively, *L*. *decora* owned more polymorphisms than *N*. *insignis* almost at each locus with the exception of several loci like *B27*, *D22* and *GA2ox1* (**Table 1**). Results of linkage equilibrium test suggested no significant deviation from linkage equilibrium between each pair of loci following Bonferroni correction (see Supplementary Note S1). In the single-locus neutral test, the mean Tajima’s *D* was significantly negative for *GA2ox1* of *N*. *insignis*, while MFDM analyses indicated that there were significant probability (*P* < 0.05) of selection acting on two loci: *A39* (*P* = 0.035) for *L*. *decora* and *GA2ox1* (*P* = 0.013) for *N*. *insignis*. When two different MDs (migration detectors) were used to analyze the possibility of migration causing unbalanced trees, both these analyses indicated migration was not responsible for unbalanced trees.

**Table 1:** Summary of characteristics, genetic parameters and neutrality tests for nuclear loci of *L*. *decora* and *N*. *insignis*.

			*L*. *decora*								*N*. *insignis*							
Locus	L	Rm	S	θ_w_× 10^-3^	π × 10^-3^	N_h_	He	Tajima’s *D*	*P*	*P* (MFDM)	S	θ_w_× 10^-3^	π × 10^-3^	N_h_	He	Tajima’s *D*	*P*	*P* (MFDM)
*A27*	541	4	12	4.18	5.98	14	0.737	1.128	0.880	0.074	4	1.31	0.64	6	0.274	-0.945	0.194	0.054
*A39*	713	0	7	1.86	0.82	6	0.273	-1.287	0.090	0.035^∗^	0	0	0	1	0	NA	NA	NA
*AroB*	591	4	7	2.24	4.02	22	0.930	1.825	0.958	0.319	4	1.20	2.25	14	0.809	1.612	0.941	0.554
*B27*	656	1	9	2.58	1.67	7	0.486	-0.866	0.209	0.106	3	0.81	0.96	6	0.556	0.299	0.696	0.113
*C12*	645	1	14	4.16	3.40	10	0.620	-0.487	0.361	0.071	4	1.10	0.48	6	0.279	-1.029	0.156	1.000
*C44*	498	4	19	7.19	5.98	24	0.908	-0.474	0.361	0.061	3	1.07	0.34	4	0.120	-1.129	0.103	1.000
*D22*	689	3	6	1.64	0.70	7	0.384	-1.261	0.090	0.071	6	1.54	2.28	10	0.730	1.007	0.857	0.403
*D34*	585	4	9	2.91	3.51	23	0.920	0.506	0.733	1.000	6	1.82	2.39	14	0.833	0.651	0.800	1.000
*GA2ox1*	869	2	11	2.38	1.53	12	0.451	-0.913	0.189	1.000	11	2.24	0.89	11	0.471	-1.474	0.034^∗^	0.013^∗^
*GAPDH*	797	5	11	2.61	2.34	13	0.777	-0.262	0.455	0.353	11	2.44	3.39	22	0.739	0.954	0.852	0.390
Mean				3.175	2.995		0.649					1.353	1.362		0.481			

*L, length; Rm, the minimum number of recombination events; S, number of segregating sites sites; θ_*w*_, Watterson’ s parameter; π: nucleotide diversity; *N*_*h*_, number of haplotypes; He, haplotype diversity; significant level, *^∗^P* < 0.05*.

### Interspecific Differentiation and Population Structure

**FIGURE 3:**
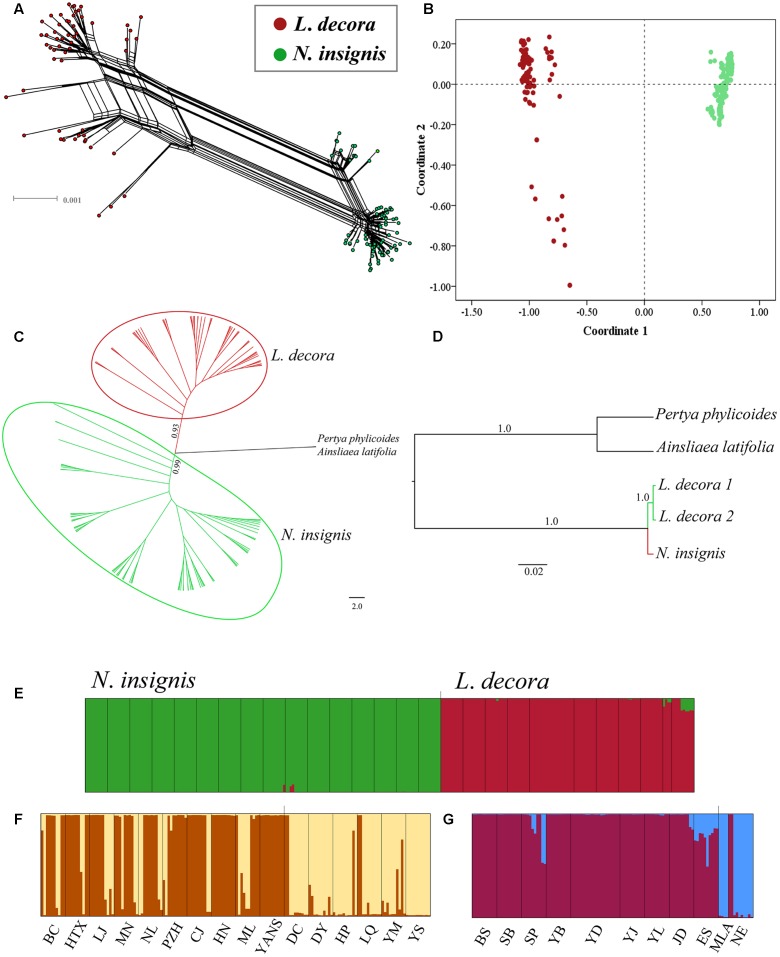
Results of the relationships between *L*. *decora* and *N*. *insignis* based on 10 nuclear DNA sequences. **(A)** Neighbor-net (N-Net) diagram illustrating relationships between all individuals. **(B)** Plot of principle component (PC) scores. **(C)** Neighbor joining phylogenetic tree of all individuals. **(D)** Species tree of all individuals inferred from ^∗^BEAST analysis. **(E)** Bayesian clustering of all *L*. *decora* and *N*. *insignis* individuals. **(F)** Bayesian clustering of all *N*. *insignis* individuals. **(G)** Bayesian clustering of all *L*. *decora* individuals. In the histograms of the STRUCTURE analyses, the smallest vertical bar represents one individual and population codes are identical to those in Supplementary Table S1.

The median joining network constructed for the haplotypes at 10 loci showed that one haplotype of the locus *C12* and *C44* was shared by *L*. *decora* and *N*. *insignis*, respectively, while other nuclear haplotypes at loci were specific to each (Supplementary Figures S2, S5). Our hierarchical AMOVA showed that genetic differentiation (F_ST_) between *L*. *decora* and *N*. *insignis* was high, ranging from the lowest 0.26 (*C44*) to the highest 0.93 (*A39*) (Supplementary Figure S3). The result from neighbor-net tree (**Figure 3A**) is congruent with those obtained in the PCoA (**Figure 3B**), resulting in samples from *L*. *decora* and *N*. *insignis* clustered into different groups, with several samples from MLA and NE most divergent in sequence. The NJ tree analysis produced similar groupings with high support (**Figure 3C**). In contrast to results obtained for the cpDNA gene tree, the species tree based on the ten nuclear loci generated by ^∗^BEAST provides strong statistical support for the two groups of *L*. *decora* as one clade with a posterior probability (PP = 1.0). *Nouelia insignis* was well supported statistically as sister clade (PP = 1.0) to *L*. *decora* (**Figure 3D**). Moreover, results of Bayesian clustering (STRUCTURE) were further interpreted for *K* = 2 when the ΔK statistic of Evanno et al. (2005) was applied (Supplementary Figure S4). Populations assigned to two clusters corresponding to a group of populations of *L*. *decora* and the other of *N*. *insignis* (**Figure 3E**). Further analyses within each of the species both showed two genetic clusters. Within *N*. *insignis*, populations (DC, DY, HP, YM, YS, and LQ) distributed in central locations were gathered in one cluster and other populations formed the other cluster (**Figure 3F**); within *L*. *decora*, individuals from two southern marginal populations MLA and NE were assigned to one cluster and others fell into the other (**Figure 3G**). Genetic admixture within two clusters in *N*. *insignis* was observed and probably reflects gene flow among nearby populations and shared ancestry due to recent divergence.

### Ecological Niche Modeling

**FIGURE 4:**
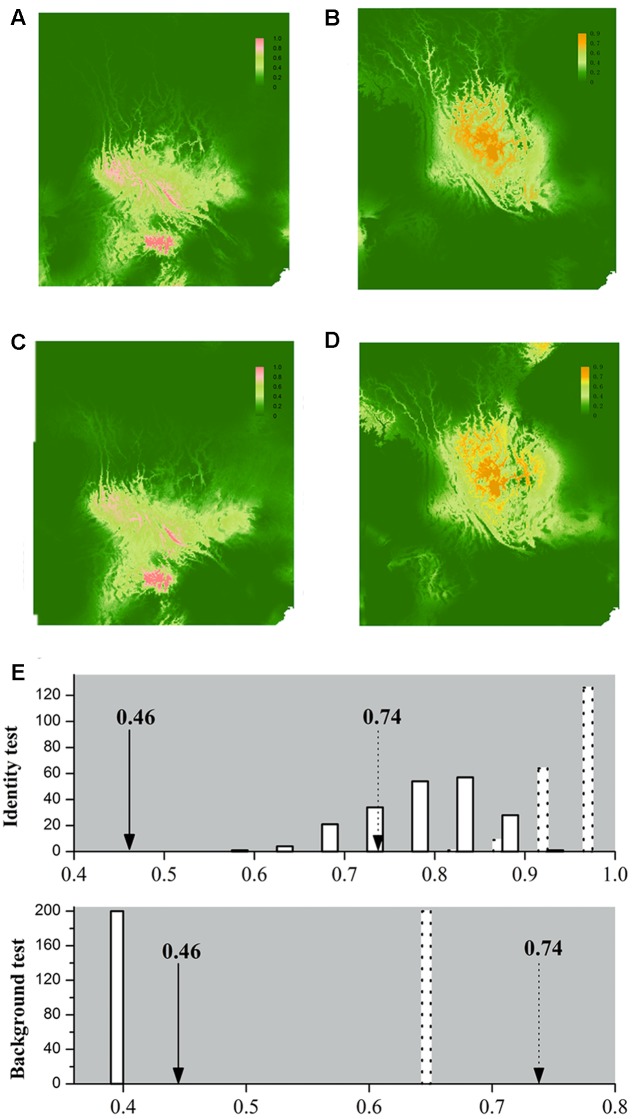
Ecological niche models (ENMs) for *L*. *decora* and *N*. *insignis* and results of the identity test and background test. Species distribution of *L*. *decora* **(A,C)** and *N*. *insignis* **(B,D)** predicted by ecological niche modeling based on seven bioclimatic variables and altitude representing the current **(A,B)** and LGM (∼21 kya, **C,D**) climatic conditions, respectively. In the result of identity test and background test **(E)**, the *x*-axis indicates values of I and D, and *y*-axis indicates number of randomizations. Null distributions are shown by solid bars for D and dotted bars for I, and arrows indicate the observed values of niche similarity (solid line for D and dotted line for I). We got the same estimation values for null distribution in two directions in the background test, therefore, the result of one direction is shown.

Our application of bioclimatic and altitude envelop modeling showed that ENMs for both species performed well. For *L*. *decora*, the average support values of AUC were 0.935 (*SD* = 0.028); for *N*. *insignis*, these values were AUC = 0.913 (*SD* = 0.040). Maxent statistics suggested that variable contributions to models were different between species (**Table 2**). The ENMs estimated the potentially suitable ecological spaces for both species that matched their present distributions (**Figures 4A,B**). The CCSM and MIROC (data not shown) models for the LGM yielded similar inferences of *L*. *decora* and *N*. *insignis* paleodistribution: the potential distribution ranges for both species contracted locally during the LGM: the ranges in southwest for *L*. *decora* showed somewhat restricted than their present ranges (**Figure 4C**), while ENM for *N*. *insignis* indicated no suitable areas in Nanpan River drainage than the current model (**Figure 4D**). Niche identity tests indicated that estimated ENMs for *L*. *decora* and *N*. *insignis* were more dissimilar than expected by chance through randomization of collection localities (*P* < 0.001) (**Figure 4E**). Background tests in both direction comparison between *L*. *decora* and *N*. *insignis* also showed that the ecological niche of *N*. *insignis* was different from that of *L*. *decora* (*P* < 0.001) (**Figure 4E**).

**Table 2:** Estimates of relatively contributions (%) of bioclimatic variables and altitude ranked according to the MAXENT models for *L*. *decora* and *N*. *insignis*, respectively.

Variables	Description	*L*. *decora*	*N*. *insignis*
bio2	Mean diurnal range	0	4.7
bio3	Isothermality	**30.4**	1.7
bio4	Temperature seasonality	11.6	**43.7**
bio7	Temperature annual range	0.1	0.4
bio13	Precipitation of wettest month	27	17.5
bio14	Precipitation of driest month	28	3.0
bio15	Precipitation seasonality	0	6.2
altitude		2.8	22.8

*Highlighted values show the highest variable contributions to models*.

### Coalescent-Based Analyses

**FIGURE 5:**
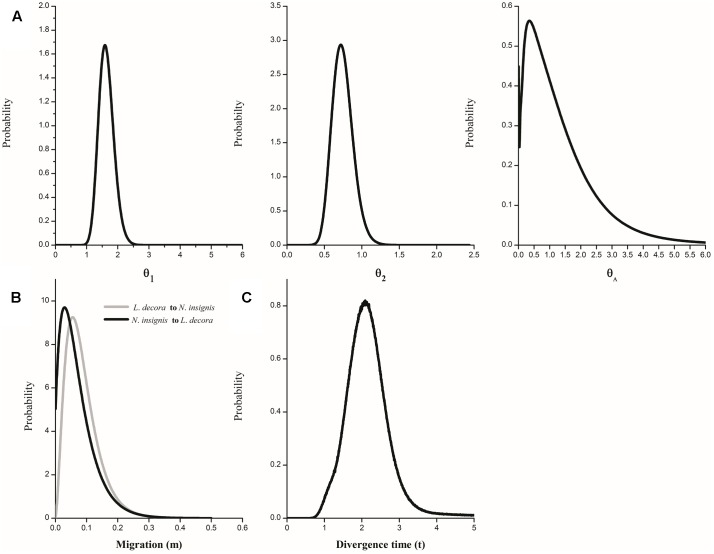
Marginal distribution of posterior probabilities for demographic parameters estimated by IM model. **(A)** Marginal distribution of the effective population sizes of *L*. *decora* (θ_1_), *N*. *insignis* (θ_2_) and their ancestral population (θ_A_); **(B)** Migration rate between *L*. *decora* and *N*. *insignis*; **(C)** The divergence time between *L*. *decora* and *N*. *insignis*.

The IMa coalescent analysis based on cpDNA dataset detected pronounced gene flow from *L*. *decora* to *N*. *insignis* but no gene flow from the opposite direction (Supplementary Figure S6A). Migration estimates from all nuclear data, although non-zero peak posterior distribution estimates of migration parameters were shown, were both very low (Supplementary Figure S6B). The parameter estimations on the basis of the combining datasets showed that the effective population size of *L*. *decora* was larger than that for *N*. *insignis*, and both were higher than that of the ancestral population (**Figure 5A**). Moreover, we found that the estimates of migration in both directions were very low (**Figure 5B**). The effective population migration rates were close to zero: 2N_1_m_1_ = 0.062 from *L*. *decora* to *N*. *insignis* and 2N_2_m_2_ = 0.008 from *N*. *insignis* to *L*. *decora*, respectively. These results were consistent with our previous study (Zhao and Gong, 2015). The posterior distribution of t peaked at about 1.758 (**Figure 5C**), which converted into a divergence time of c. 2.068 Mya (90% HPD interval: 1.16-2.91 Mya) based on the geometric average mutation rate.

In ABC models, the highest posterior probability was found for scenario 1 (0.9987, 95% CIs: 0.9981–0.9992), and much higher than for scenario 2 (0.0013, 95% CIs: 0.0008–0.0019). The result suggested that *L*. *decora* and *N*. *insignis* diverged from the common ancestor and *N*. *insignis* experienced bottlenecks during the glacial period (Supplementary Figure S7 and Supplementary Table S5). Assuming 4–5 years as generation time, the divergence time scaled to 2.34 (95% CIs: 1.5–2.8 Ma)-2.925 Ma (95% CIs: 1.87–3.47 Ma) and the bottleneck event was dated from around 80 to 105 ka. The median values of effective population size of *L*. *decora* and *N*. *insignis* were 109,000 (95% CIs: 56,600–176,000) and 52,400 (95% CIs: 23,700–95,100), respectively; and in comparison with those of *L*. *decora* and *N*. *insignis*, the median value of effective population size for the ancestral population was much lower (Supplementary Figure S7 and Supplementary Table S5), suggesting expansion events after their divergence. All observed summary statistics were not significantly different from the simulated values based on parameter values drawn from the posterior distributions for scenario 1. The type I error for scenario 1 was very low (4.2%), and the type II errors for scenario 1 to scenario 2 was 29.1%.

## Discussion

### Interspecific Relationship and Divergence between *L. decora* and *N. insignis*

Previous phylogenetic analysis on major clades of the Asteraceae revealed that *Nouelia* and *Leucomeris* was sister to each other (Panero and Funk, 2008). However, interspecific relationships within the two genera have not been well resolved. Our phylogenetic investigation using ten cytoplasmic markers as those of Panero and Funk (2008), showed that all haplotypes of *L*. *decora* and *N*. *insignis* was assigned to a monophyletic clade, supporting their closer relationship in maternal lineage than that between *L*. *decora* and *L*. *spectabilis*. However, within the monophyletic clade, all the haplotypes of *N*. *insignis* were nested with one clade of *L*. *decora* without forming a clear monophyly, indicating the recent divergence between the two species (**Figure 1**). In contrast to the phylogenetic pattern of the chloroplast gene trees, multiple pieces of genetic evidence (e.g., species tree, multi-locus network, Structure) provide support to delimit *L. decora* from *N. insignis*, with the two main clade/clustering corresponding to the recognized taxonomy (**Figure 3**).

Such conflict of cytoplasmic-nuclear discordance might be attributed to the incomplete lineage sorting (Jakob and Blattner, 2006) or introgressive hybridization (Abbott et al., 2013). Here, the explanation of incomplete lineage sorting seems unlikely, given the observed phylogeographic pattern as well as due to the fact that it would take in average more generations for the nuclear DNA of a lineage to coalesce into common ancestor back in time and reach monophyly than that of chloroplast DNA (i.e., complete lineage sorting through drift) (e.g., Qi et al., 2012). Instead, we argued that introgression of chloroplast DNA rather than incomplete lineage sorting should be referred as an important explanation that caused the conflicting placement of haplotypes from *N*. *insignis*. The idea is also supported by our simulation under the isolation with migration (IM) model that identified significant signal of chloroplast DNA gene flow from *L*. *decora* to *N*. *insignis* (Supplementary Figure S6A). However, our analyses are unable to determine whether the gene flow occurred during speciation (e.g., under conditions of parapatry) or following secondary contact after initial allopatric divergence. Considering that the cytoplasmic-nuclear discordance was observed in central but marginal populations such as SB and SP of *L*. *decora*, it is more likely attributed to the result of chloroplast DNA introgression during their expansion without major geographical barriers, since the coalescent-based analyses unambiguously showed that their effective population size are both larger than that of the ancestor after divergence (**Figure 5A** and Supplementary Table S5). Moreover, given the morphological characters such as their achenes bearing abundant pappi, it could be inferred that the seed flow is more possibly efficient than pollen flow. Such situation has been observed in other species in Asteraceae (e.g., Wang J.F. et al., 2013).

### Pliocene/Pleistocene Divergent Event in *L. decora* and *N. insignis*

Our DIYABC results estimated from multiple low copy nuclear genes showed the divergence of the two species from a common ancestor at *c*. 2.34–2.925 Ma (average 2.63 Ma), with an assuming generation time of 4–5 years. Moreover, the dating of divergence events was further examined using Isolation with Migration (IMa) based on multiple nuclear genes, and the result suggest a split between the two species at *c*. 2.068 Mya (**Figure 5C**). The nearly congruent time estimated from multiple genes increases the reliability that it correctly reflects the divergent event between the two species. Supporting evidence of diversification along the ‘Tanaka Line’ of Southwest China flora, was only found in a limited number of phylogeographic studies. However, most of these studies suggested intraspecific divergences profoundly affected by the Mid/Late Pleistocene climatic oscillations in this area, interspecific divergence during the Pliocene/ Early-Pleistocene was seldom revealed (Zhang et al., 2006; Fan et al., 2013; Tian et al., 2015; Zhao and Zhang, 2015). Our estimated divergence time indicates the divergence may be closely associated with the climatic transformation from Pliocene to Pleistocene. During Pliocene, Yunnan plateau was under the setting of weak tectonic movement, and the relatively flat terrain and warm-humid climate might favor the spread of ancestor of *Nouelia* and *Leucomeris* into the Yunnan plateau possibly from Indochina Peninsula, where they were widely distributed in tropical forest (Li, 1995). However, strong tectonic movement in the Yunnan plateau was caused by the accelerated uplift of the QTP during the late Pliocene (Cheng et al., 2001). In the mean time, the climate transformed from a warm and wet to colder and drier in southwest China due to the East Asian monsoon shift (Yao et al., 2012; Su et al., 2013), especially the temperature declined for both summer and winter (Huang et al., 2015). If diversification was triggered mainly by geological event (uplifting of Yunnan Plateau) related to the formation of large mountains ranges and river systems, the onset of diversification need to be conditioned by the presence of geographic barriers. However, the particularly propound barrier to dispersal along the ‘Tanaka Line’ is not found (Li and Li, 1992, 1997), the divergence associated with the biogeographical boundaries is more likely attributed to the ecological processes (Glor and Warren, 2011), in which diversification was driven by climatic fluctuation of this area.

### Evolutionary History of *N. insignis*

According to the results of genetic differentiation and divergence time estimations, it can be proposed that the climatic shift from the Late Pliocene to Pleistocene have acted as a stimulus to promote divergence of *N*. *insignis* and *L*. *decora* from their common ancestor (Li, 1995). Notably, population divergence or speciation possibly triggered by Pleistocene climate change has also been indicated in other flora in subtropical China, such as *Pinus Yunnanensis* (Wang B. et al., 2013) and *Dysosma versipeuis*-*pleiantha* complex (Wang et al., 2017), in which the importance of ecological factors in forming or maintaining genetic divergence was demonstrated (see discussion below). If taking the factor of environmental change associated with the tectonic event into consideration, the impact of altitude on the divergence should not be ignored, as environmental gradients associated with altitude can facilitate niche divergence (Korner, 2007). The geological studies has shown that during the late Pliocene, the affecting strength by uplift of the QTP on Yunnan Plateau, gradually weakened from northwest to southeast, resulting in significant altitude differences within this area. The ancestor *L*. *decora* might survive in lowerelevation, whereas those distributed with high altitudes might be suffering from the deteriorating climatic conditions and started to diversify in the Early Pleistocene.

Actually, the scenario is further strengthened by our niche modeling, which showed their differences in preference of climate condition and suggested they occupy significantly different climatic environments (**Figure 4E**). For *N*. *insignis*, it is sensitive to altitude and seasonal climate stability (bio4), whereas the temperature changes (bio3) and precipitation of wettest (bio16) and driest (bio17) Quarter are dominant factors impacting on the distribution of *L*. *decora* (**Table 2**). Further, we found that when randomized seedlings of *N*. *insignis* was transplanted into the botany garden, they can live to reproduce for about 4–5 years; however, failure was detected for those of *L*. *decora* in the common garden. This partially implied divergent selection between environments and local adaptation to their respective habitats, although these could not represent the whole reciprocal transplant experiment. The significant niche differentiation, together with the chloroplast DNA haplotype distribution pattern, suggests that *N*. *insignis* might have originated in the central Yunnan Plateau and then migrated to northwest, where almost all populations were completely fixed by cpDNA haplotypes (H3) (**Figure 2**). During the process, it tended to migrate toward lower altitudes from high mountain to lower valleys controlled by foehn with little annual raining (Jin, 1999) and lower latitudes after the LGM period (such as Nanpan River) (**Figures 4B,D**) to track optimal ecological conditions with the decrease of temperature. Nevertheless, the condition in Nanpan River drainage is much wetter, resulting in seed reproduction and seedling growing partly inhibited (Peng et al., 2003), which in turn reflect precipitation plays an important role in its adaptation. In consequence, it ultimately has distribution ranges confined to the dry valley, which is the most representative of its habitat.

### Divergence with Gene Flow between *L. decora* and *N. insignis*

Empirical investigations have demonstrated that species divergence in the face of gene flow is feasible under particular circumstances, when natural divergent selection could overcome homogenizing effects of gene flow, and ultimately generate distinct gene pools (Coyne and Orr, 2004). Here, in accordance with recent findings (Li et al., 2010; Harrington et al., 2013; Wang Q. et al., 2013; Sun et al., 2016), we present another case of divergence with gene flow. This suggested that the maintenance of the two species despite the existence of gene flow might be the consequence of adaptive divergence. Our results of *F*_ST_ for *L*. *decora* and *N*. *insignis* showed that the interspecific *F*_ST_ values were highest for *A39* (0.93) and secondly for *GA2ox1* (0.89) (Supplementary Figure S3). The possible mechanism for this genetic differentiation pattern is that divergent selection itself can promote genetic differentiation through increasing genetic differentiation of regions affected by selection (Nosil et al., 2009). Additionally, we also noted that heterogeneous gene divergence existed between *L*. *decora* and *N*. *insignis*. Take the gene *A39* as an example, the genealogic diagram showed that one haplotype tends to be fixed in *N*. *insignis*, while *L*. *decora* shows a more diverse pattern in this locus (Supplementary Figure S5). This observation is in accordance with the hypothesis that divergence selection on one locus can strongly affected the frequency of alleles at selected loci (Nosil et al., 2009; Feder et al., 2012). Furthermore, two different approaches of the neutral tests, one (MFDM) of which has shown high power for detecting recent positive selection and is widely used recently, were conducted. Encouragingly, the loci detected by both methods almost overlapped. Therefore, these results together indicated that the two nuclear loci (*A39* and *GA2ox1*) might be subjected to past divergent selection within *L*. *decora* and *N*. *insignis*, respectively.

*GA2ox1* (gibberellin 2-oxidase 1) is one of the key enzymes in GA biosynthesis and metabolism, and often utilized for dwarf plant engineering breeding (Ross et al., 1995; El-Sharkawy et al., 2012). The species, *N*. *insignis* now mainly distributed in dry-hot valleys, might have experienced stronger water stress and has a dwarf growth form, which correspond to the increased divergence at the *GA2ox1* locus in this species. Although the function of *A39* playing in the two species is not clear, it is possible that the selection for different alleles might reflect adaptation to their respective habitats. Among the ten nuclear loci, only two loci have been detected to be subject to past divergent selection. Similar proportion of loci under possible divergent selection have been also observed in other species, such as birds (Li et al., 2010) and woody plants (Li et al., 2011). These divergent population genetic studies have demonstrated divergent selection existed among the specific loci. Similar result inferred from our datasets is in agreement with that diversifying selection often acts on specific genome regions, while others are showing low levels of differentiation (Hey, 2006; Nosil et al., 2009). If more extensive genome sampling is conducted, the proportion of genome-wide divergent selection may be identified and assessed among these two species.

## Conclusion

Numerous studies have revealed that geographic isolation could not adequately explain speciation and diversification, but factors such as local selection may likely and prevalently promoted divergence of closely related species. This study explored the divergence of two closely related species distributed across the two sides of the ‘Tanaka Line’ and revealed the divergence was primarily triggered by the climatic shift from Pliocene to Pleistocene, which support the ecological factors driving species differentiation. Despite the deep divergence of nuclear genes, the chloroplast DNA gene flow seems not to be interrupted completely. The significant ecological differentiation and the two loci discovered under divergent selection supported the two species experienced divergent selection since their differentiation, which may act as a role to overcome the homogenizing effect of gene flow. Our study provides an additional case of divergence with gene flow closely correlated with climatic shifts and understanding what conditions might have promoted the extraordinary biodiversity in southwest China.

## Author Contributions

YZ and XG planned and designed the research. GY and YP conducted field work and figures made. YZ performed the experiments and analyzed the data. YZ and XG wrote the manuscript.

## Conflict of Interest Statement

The authors declare that the research was conducted in the absence of any commercial or financial relationships that could be construed as a potential conflict of interest.

## Footnotes

1 http://www.fluxus-engineering.com

2 http://www.cvh.ac.cn/
