# Supplementary material for: Ecological and Genetic Divergences with Gene Flow of Two Sister Species (Leucomeris decora and Nouelia insignis) Driving by Climatic Transition in Southwest China
Source: Front Plant Sci. 2018 Jan 25;9:31. doi: 10.3389/fpls.2018.00031 (PMC5789531; doi:10.3389/fpls.2018.00031)
Supplement: Supplementary file 1 [file Presentation_1.PDF]

Supplementary Material

Ecological and genetic divergences with gene flow of two sister species (*Leucomeris decora* and *Nouelia insignis*) driving by climatic transition in Southwest China

Yujuan Zhao, Genshen Yin, Yuezhi Pan, Xun Gong*

Correspondence:

*Xun Gong

E-mail: gongxun@mail.kib.ac.cn

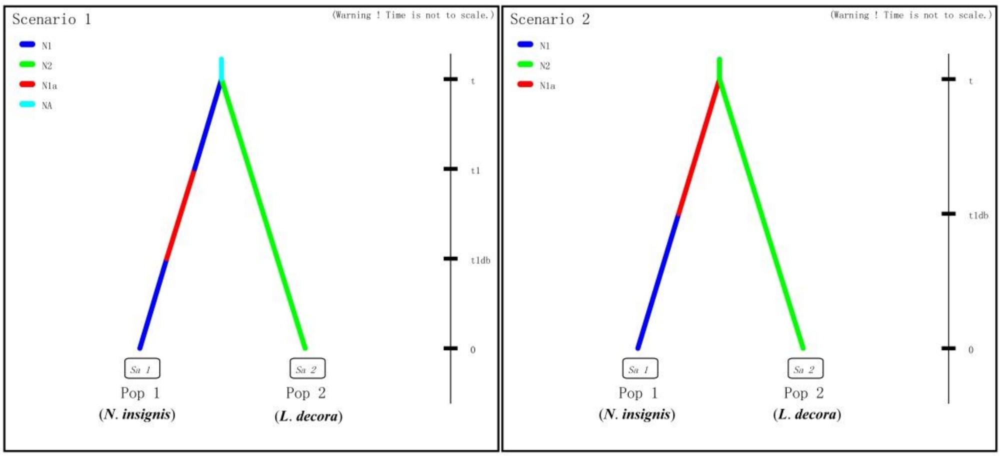

Figure S1 Scenarios of divergence between *L. decora* and *N. insignis* tested using approximate Bayesian computation (ABC). In these scenarios, t# represents time scale in terms of the number of generations and N# represents the effective population size of the different populations.

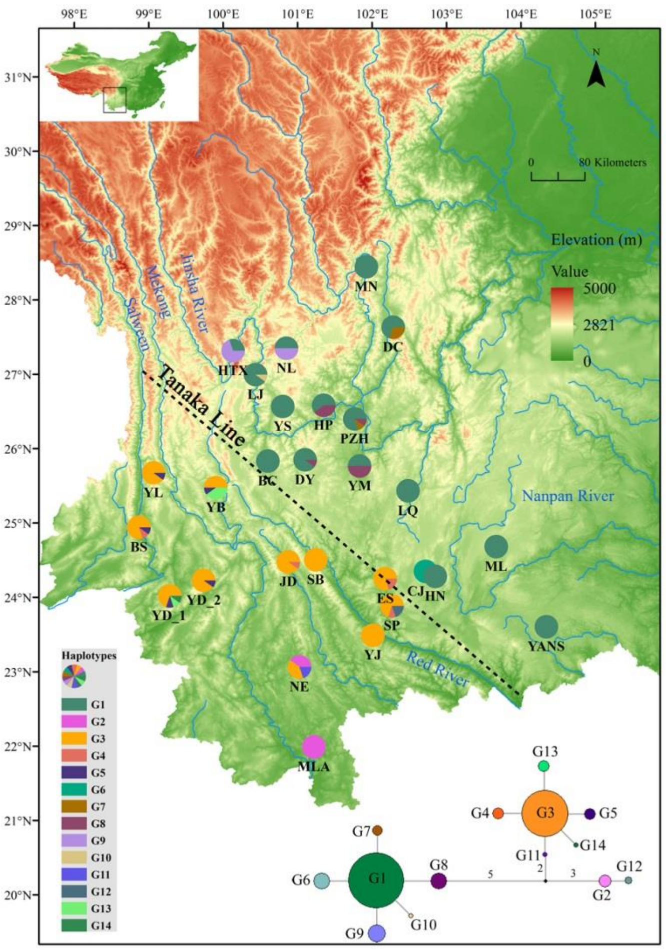

Figure S2 Geographical distribution and network of nuclear (*GA2ox1*) haplotypes (G1-G14) without recombination identified in 28 populations of *L. decora* and *N. insignis*. The dash line shows the location of 'Tanaka Line'.

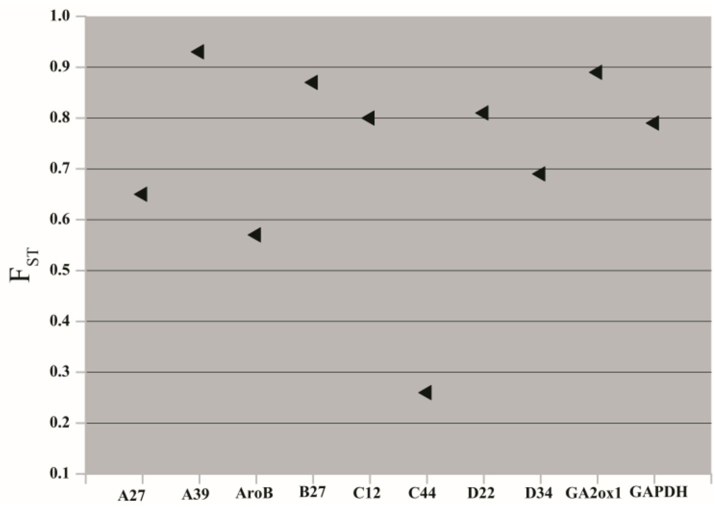

Figure S3 Results of F_{ST} for *L. decora* and *N. insignis* pairs. The x-axis indicates the nuclear gene, and y-axis indicates values of F_{ST}. The triangles indicate the values of F_{ST} for each nuclear gene.

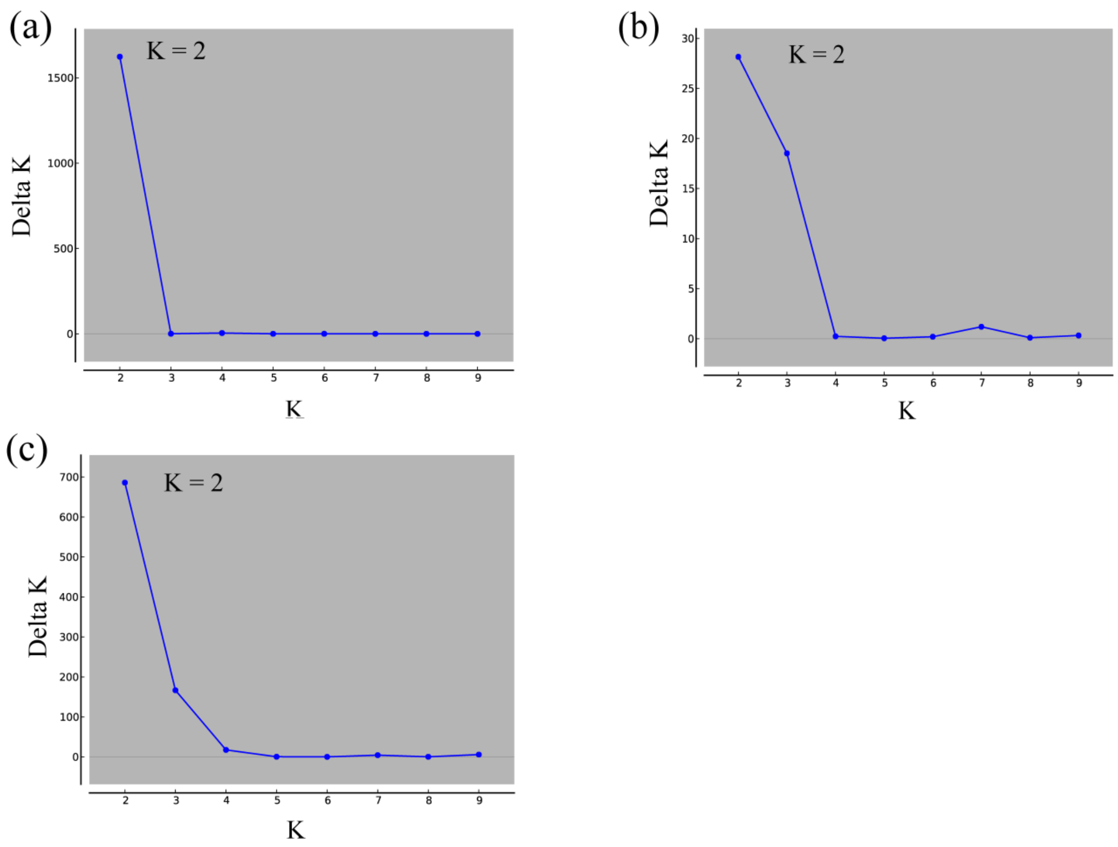

Figure S4 Results of Bayesian clustering analyses in STRUCTURE considering (a) *L. decora* and *N. insignis*, (b) *L. decora*, (c) *N. insignis*. Diagram showing the optimal number of groups (K) obtained by across using ΔK statistic of Evanno *et al.* (2005).

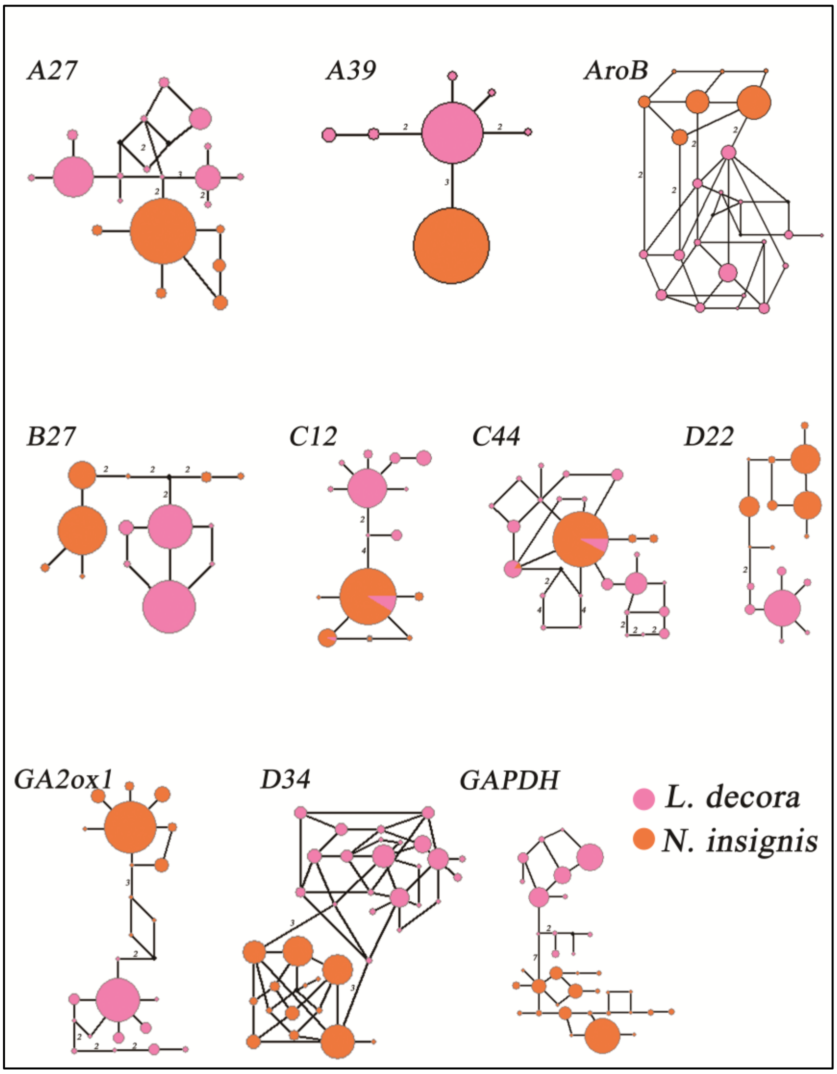

Figure S5 Networks of haplotypes constructed using NETWORK for 10 nuclear DNA loci.

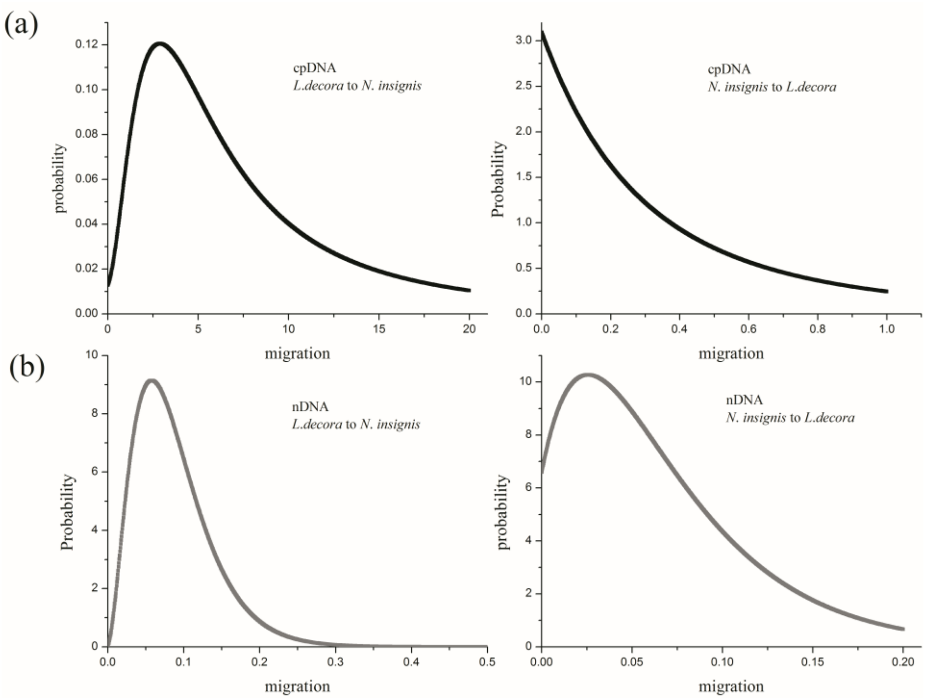

Figure S6 Marginal distribution of posterior probability for migration rate between *L. decora* and *N. insignis* based on chloroplast (a) and nuclear DNA (b) markers, respectively.

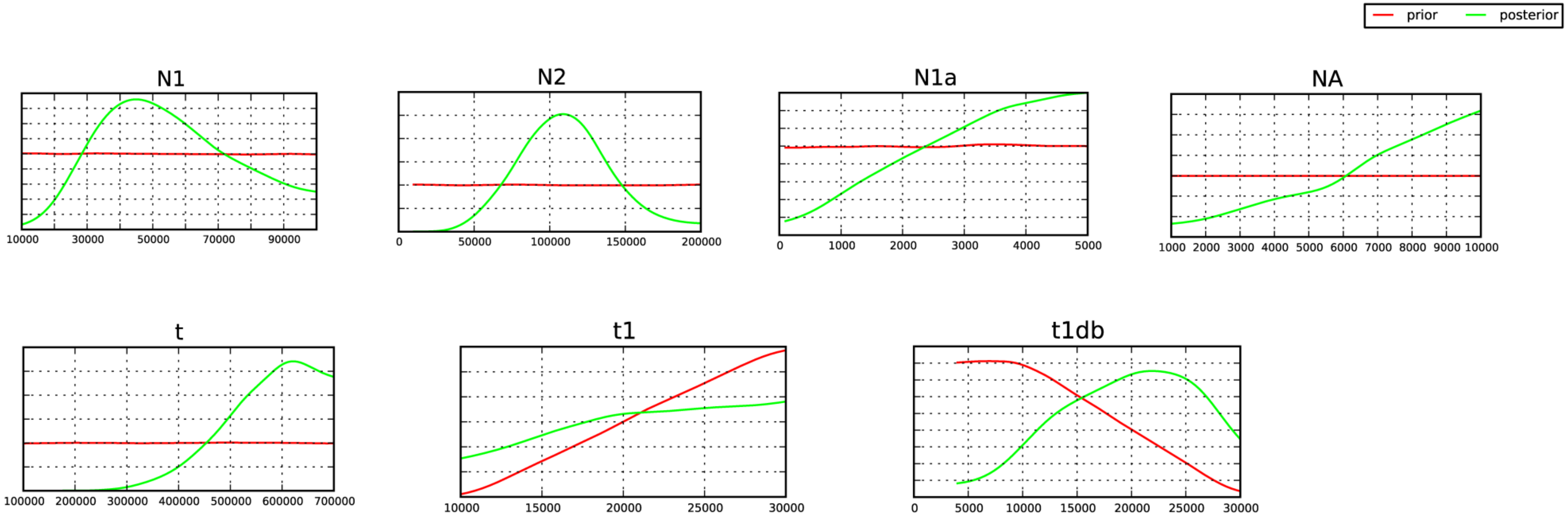

Figure S7 Estimations of the prior and posterior distributions of parameters revealed by DIY-ABC modeling of the best scenario for the demographic history of *L. decora* and *N. insignis*. Time (t) is in generations with each generation equivalent to five years.

Table S1 Collection information for 12 populations of *L. decora* and 16 populations of *N. insignis* sampled in this study.

Population code	Location	Latitude (°N)	Longitude (°E)	Elevation (m)	Samples (n)
L.decora					
MLA	Mengla, Yunnan	21.990	101.220	1440	2
NE	Ninger, Yunnan	23.065	101.026	1800	5
YJ	Yuanjiang, Yunnan	23.490	102.011	1450	5
SP	Shiping, Yunnan	23.886	102.267	1600	5
ES	Eshan, Yunnan	24.252	102.177	1470	5
YD-1	Yongde-1, Yunnan	24.021	99.284	1680	5
YD-2	Yongde-2, Yunnan	24.230	99.738	1480	5
JD	Jingdong, Yunnan	24.478	100.875	1580	5
BS	Baoshan, Yunnan	24.943	98.868	1510	5
YB	Yangbi, Yunnan	25.476	99.902	1390	5
YL	Yunlong, Yunnan	25.675	99.065	1790	5
SB	Shuangbai,Yunnan	24.505	101.243	1500	5
N.insignis					
BC	Dali, Yunnan	25.833	100.600	1510	5
DC	Dechang, Sichuan	27.633	102.283	1860	5
DY	Dayao, Yunnan	25.850	101.100	1500	5
HP	Huaping, Yunnan	26.583	101.350	2120	5
HTX	Zhongdian, Yunnan	27.317	100.133	1988	5
LJ	Lijiang, Yunnan	26.995	100.435	2400	5
LQ	Luquan,Yunnan	25.433	102.483	1650	5
MN	Mianning, Sichuan	28.447	101.923	1600	5
NL	Ninglang, Yunnan	27.350	100.85	2200	5
PZH	Panzhihua, Sichuan	26.400	101.767	1210	5
YM	Yuanmou,Yunnan	25.767	101.833	2040	5
YS	Yongsheng,Yunnan	26.567	100.800	1670-1910	5
CJ	Chengjiang, Yunnan	24.350	102.717	1988	5
HN	Huaning, Yunnan	24.283	102.850	1450	5
ML	Mile, Yunnan	24.683	103.667	1900	5
YANS	Yanshan, Yunnan	23.606	104.341	1540	5
total					137

Table S2 Sequences information of chloroplast DNA from NCBI used for phylogenetic analysis.

matK	>gi|170665391|gb|EU385336.1| *Chimantaea humilis maturase* K (matK) gene
	>gi|170665453|gb|EU385367.1| *Hyalis argentea* maturase K (matK) gene
	>gi|170665457|gb|EU385369.1| *Ianthopappus corymbosus* maturase K (matK) gene
	>gi|170665463|gb|EU385372.1| *Leucomeris spectabilis* maturase K (matK) gene
	>gi|170665515|gb|EU385398.1| *Stenopadus talaumifolius* maturase K (matK) gene
	>gi|170665521|gb|EU385401.1| *Stomatochaeta condensata* maturase K (matK) gene
	>gi|170665534|gb|EU385408.1| *Wunderlichia mirabilis* maturase K (matK) gene
ndhD	>gi|170665199|gb|EU385240.1| *Chimantaea humilis* NADH dehydrogenase subunit D (ndhD) gene
	>gi|170665261|gb|EU385271.1| *Hyalis argentea* NADH dehydrogenase subunit D (ndhD) gene
	>gi|170665265|gb|EU385273.1| *Ianthopappus corymbosus* NADH dehydrogenase subunit D (ndhD) gene
	>gi|170665271|gb|EU385276.1| *Leucomeris spectabilis* NADH dehydrogenase subunit D (ndhD) gene
	>gi|170665325|gb|EU385303.1| *Stenopadus talaumifolius* NADH dehydrogenase subunit D (ndhD) gene
	>gi|170665331|gb|EU385306.1| *Stomatochaeta condensata* NADH dehydrogenase subunit D (ndhD) gene
	>gi|170665345|gb|EU385313.1| *Wunderlichia mirabilis* NADH dehydrogenase subunit D (ndhD) gene
ndhF	>gi|186885634|gb|EU385144.1| *Chimantaea humilis* NADH dehydrogenase subunit F (ndhF) gene
	>gi|186885696|gb|EU385175.1| *Hyalis argentea* NADH dehydrogenase subunit F (ndhF) gene
	>gi|186885700|gb|EU385177.1| *Ianthopappus corymbosus* NADH dehydrogenase subunit F (ndhF) gene
	>gi|186885706|gb|EU385180.1| *Leucomeris spectabilis*NADH dehydrogenase subunit F (ndhF) gene
	>gi|186885760|gb|EU385207.1| *Stenopadus talaumifolius* NADH dehydrogenase subunit F (ndhF) gene
	>gi|186885766|gb|EU385210.1| *Stomatochaeta condensata* NADH dehydrogenase subunit F (ndhF) gene
	>gi|186885780|gb|EU385217.1| *Wunderlichia mirabilis* NADH dehydrogenase subunit F (ndhF) gene
ndhI	>gi|168188246|gb|EU243263.1| *Chimantaea humilis* NADH dehydrogenase subunit I (ndhI) gene
	>gi|168188304|gb|EU243293.1| *Hyalis argentea* NADH dehydrogenase subunit I (ndhI) gene
	>gi|168188308|gb|EU243295.1| *Ianthopappus corymbosus* NADH dehydrogenase subunit I (ndhI) gene
	>gi|168188314|gb|EU243298.1| *Leucomeris spectabilis*NADH dehydrogenase subunit I (ndhI) gene
	>gi|168188363|gb|EU243323.1| *Stenopadus talaumifolius* NADH dehydrogenase subunit I (ndhI) gene
	>gi|168188369|gb|EU243326.1| *Stomatochaeta condensata* NADH dehydrogenase subunit I (ndhI) gene
	>gi|168188382|gb|EU243333.1| *Wunderlichia mirabilis* NADH dehydrogenase subunit I (ndhI) gene
rbcL	>gi|170664919|gb|EU384958.1| *Chimantaea humilis* ribulose 1,5-bisphosphate carboxylase/oxygenase large subunit (rbcL) gene
	>gi|170664979|gb|EU384988.1| *Hyalis argentea* ribulose 1,5-bisphosphate carboxylase/oxygenase large subunit (rbcL) gene
	>gi|170664983|gb|EU384990.1| *Ianthopappus corymbosus* ribulose 1,5-bisphosphate carboxylase/oxygenase large subunit (rbcL) gene
	>gi|170664989|gb|EU384993.1| *Leucomeris spectabilis*ribulose 1,5-bisphosphate carboxylase/oxygenase large subunit (rbcL) gene
	>gi|170665041|gb|EU385019.1| *Stenopadus talaumifolius* ribulose 1,5-bisphosphate carboxylase/oxygenase large subunit (rbcL) gene
	>gi|170665045|gb|EU385021.1| *Stomatochaeta condensata* ribulose 1,5-bisphosphate carboxylase/oxygenase large subunit (rbcL) gene
	>gi|170665059|gb|EU385028.1| *Wunderlichia mirabilis* ribulose 1,5-bisphosphate carboxylase/oxygenase

	large subunit (rbcL) gene
rpoB	>gi|186885826|gb|EU385431.1| *Chimantaea humilis* RNA polymerase beta subunit (rpoB) gene
	>gi|186885888|gb|EU385462.1| *Hyalis argentea* RNA polymerase beta subunit (rpoB) gene
	>gi|186885892|gb|EU385464.1| *Ianthopappus corymbosus* RNA polymerase beta subunit (rpoB) gene
	>gi|186885898|gb|EU385467.1| *Leucomeris spectabilis*RNA polymerase beta subunit (rpoB) gene
	>gi|186885952|gb|EU385494.1| *Stenopadus talaumifolius* RNA polymerase beta subunit (rpoB) gene
	>gi|186885958|gb|EU385497.1| *Stomatochaeta condensata* RNA polymerase beta subunit (rpoB) gene
	>gi|186885972|gb|EU385504.1| *Wunderlichia mirabilis* RNA polymerase beta subunit (rpoB) gene
rpoC	>gi|170665580|gb|EU385527.1| *Chimantaea humilis* RNA polymerase beta' subunit (rpoC1) gene
	>gi|170665640|gb|EU385557.1| *Hyalis argentea* RNA polymerase beta' subunit (rpoC1) gene
	>gi|170665644|gb|EU385559.1| *Ianthopappus corymbosus* RNA polymerase beta' subunit (rpoC1) gene
	>gi|170665654|gb|EU385564.1| *Leucomeris spectabilis*RNA polymerase beta' subunit (rpoC1) gene
	>gi|170665708|gb|EU385591.1| *Stenopadus talaumifolius* RNA polymerase beta' subunit (rpoC1) gene
	>gi|170665714|gb|EU385594.1| *Stomatochaeta condensata* RNA polymerase beta' subunit (rpoC1) gene
	>gi|170665728|gb|EU385601.1| *Wunderlichia mirabilis* RNA polymerase beta' subunit (rpoC1) gene
trnA	>gi|165928695|gb|EU243168.1| *Chimantaea humilis* 23S ribosomal RNA-trnA intergenic spacer and tRNA-Ala (trnA) genes
	>gi|165928726|gb|EU243199.1| *Hyalis argentea* 23S ribosomal RNA-trnA intergenic spacer and tRNA-Ala (trnA) genes
	>gi|165928728|gb|EU243201.1| *Ianthopappus corymbosus* 23S ribosomal RNA-trnA intergenic spacer and tRNA-Ala (trnA) genes
	>gi|165928731|gb|EU243204.1| *Leucomeris spectabilis*23S ribosomal RNA-trnA intergenic spacer and tRNA-Ala (trnA) genes
	>gi|165928756|gb|EU243229.1| *Stenopadus talaumifolius* 23S ribosomal RNA-trnA intergenic spacer and tRNA-Ala (trnA) genes
	>gi|165928759|gb|EU243232.1| *Stomatochaeta condensata* 23S ribosomal RNA-trnA intergenic spacer and tRNA-Ala (trnA) genes
	>gi|165928765|gb|EU243238.1| *Wunderlichia mirabilis* 23S ribosomal RNA-trnA intergenic spacer and tRNA-Ala (trnA) genes
trnL-F	>gi|170665084|gb|EU385051.1| *Chimantaea humilis* tRNA-Leu (trnL) gene and trnL-trnF intergenic spacer
	>gi|170665114|gb|EU385081.1| *Hyalis argentea* tRNA-Leu (trnL) gene and trnL-trnF intergenic spacer
	>gi|170665116|gb|EU385083.1| *Ianthopappus corymbosus* tRNA-Leu (trnL) gene and trnL-trnF intergenic spacer
	>gi|170665119|gb|EU385086.1| *Leucomeris spectabilis*tRNA-Leu (trnL) gene and trnL-trnF intergenic spacer
	>gi|170665146|gb|EU385113.1| *Stenopadus talaumifolius* tRNA-Leu (trnL) gene and trnL-trnF intergenic spacer
	>gi|170665149|gb|EU385116.1| *Stomatochaeta condensata* tRNA-Leu (trnL) gene and trnL-trnF intergenic spacer
	>gi|170665155|gb|EU385122.1| *Wunderlichia mirabilis* tRNA-Leu (trnL) gene and trnL-trnF intergenic spacer

Table S3 Locus information and list of the nuclear primers used in this study.

Locus	Putative function	PCR primers (5'-3')	Reference
AroB	Putative 3-dehydroquinate synthase activity	F: GCTTCTTCTTGCCTGATGCT<br>R:GCTTTGTTTTCACATGAWCKCTTDATAGCA	(Li *et al.*, 2008)
GA2ox1	gibberellin 2-oxidase 1	F: TTCTCYGGRTAGTTRAGATCATCAA<br>R: TTCTCGCTCAATGGTGGTCCT	(Mitsui & Setoguchi, 2012)
GAPDH	glyceraldehyde 3-phosphate dehydrogenase	F: ATCATTCCCAGCAGYACT<br>R: GCATCAGCAGAATTAGAAGG	(Zhao & Gong, 2012)
A27	unknown	F: CTTGCAWTGAATGTCATGTGGAAG<br>R: GCTCCCCARCATTTCA	(Chapman *et al.*, 2007)
A39	unknown	F: ACTAGTTGGCATYTRATGGTAACA<br>R: GCCRACAAAATTGAGCTGAAGATC	(Chapman *et al.*, 2007)
B27	unknown	F: AAGGCTCTTATGGCHATGCC<br>R: CGGTTYTTRGCWGCTTCATCCCARAACTG	(Chapman *et al.*, 2007)
C12	unknown	F: TCTTGCACCACCAACTGYTTGGC<br>R: GACACCGCCTTGGCTGC	(Chapman *et al.*, 2007)
C44	unknown	F: TGTGAAGTCTCYTTGATTCTTG<br>R: AACTTGTCTAACRGCATCTGG	(Chapman *et al.*, 2007)
D22	unknown	F: GCATCAGCAGAATTAGAAGG<br>R:GCTTCTTCTTGCCTGATGCT	(Chapman *et al.*, 2007)
D34	unknown	F: GTSCAATCWCACATWCAAATMAATGA<br>R: TTCTCYGGRTAGTTRAGATCATCAA	(Chapman *et al.*, 2007)

Table S4 Prior distribution for the demographic parameters that were applied in the DIYABC program. Uniform distribution (UN) with 2 parameters: min and max; Effective population size for *L. decora* (N1); Effective population size for *N. insignis* (N2); Ancestral Effective population size (NA); Effective population size for *N. insignis* after bottleneck (N1a); Divergence time (t); Population bottleneck time of *N. insignis* (t1); Recent population expansion time of *N. insignis* (t1db).

Parameters	Distribution
N1	UN~[1E4-1E5]
N2	UN~[1E4-2E5]
NA	UN~[1E3-1E4]
N1a	UN~[1E2-5E3]
t	UN~[1E5-7E5]
t1	UN~[1E4-3E4]
t1db	UN~[4E3-3E4]
Constraint for parameter	N2 > N1a; N1 > N1a; t > t1; t1 > t1db
Marker parameters	
mean μ	UN~[1.00E-9-1.00E-8]
gam μ	GA~[1.00E-9-1.00E-6, 2]
mean-K-C/T	UN~[0.05, 20]
gam- K-C/T	GA~[0.05, 20, 2]

Table S5 Estimations of posterior distribution of parameters revealed by DIY-ABC for the best scenario of demographic history of *L. decora* and *N. insignis* based on the nuclear multilocus sequence data

Parameter	NA	N1	N2	N1a	t (generations)	t1 (generations)	t1db (generations)
Mean	5.97E+003	5.50E+004	1.10E+005	3.22E+003	5.71E+005	2.08E+004	1.94E+004
Median	6.26E+003	5.24E+004	1.09E+005	3.40E+003	5.85E+005	2.10E+004	1.99E+004
Mode	9.62E+003	4.59E+004	1.05E+005	4.63E+003	6.04E+005	2.95E+004	2.05E+004
95% CI	1.30E+003	2.37E+004	5.66E+004	7.54E+002	3.74E+005	1.08E+004	7.53E+003
	9.85E+003	9.51E+004	1.76E+005	4.92E+003	6.93E+005	2.96E+004	2.86E+004

NA: ancestral population size; N1: current population size of *N. insignis*; N2: current population size of *L. decora*; N1a: ancestral population size of *N. insignis* during the bottleneck; t: divergence time between *L. decora* and *N. insignis* in generations; t1: the beginning time in generations of the bottleneck period for *N. insignis*; t1db: the end time in generations of the bottleneck period for *N. insignis*.

REFERENCES

Chapman, M.A., Chang, J., Weisman, D., Kesseli, R.V. & Burke, J.M. (2007) Universal markers for comparative mapping and phylogenetic analysis in the Asteraceae (Compositae). *Theoretical and Applied Genetics*, **115**, 747-755.

Li, M.G., Wunder, J., Bissoli, G., Scarponi, E., Gazzani, S., Barbaro, E., Saedler, H. & Varotto, C. (2008) Development of COS genes as universally amplifiable markers for phylogenetic reconstructions of closely related plant species. *Cladistics*, **24**, 727-745.

Mitsui, Y. & Setoguchi, H. (2012) Demographic histories of adaptively diverged riparian and non-riparian species of *Ainsliaea* (Asteraceae) inferred from coalescent analyses using multiple nuclear loci. *BMC Evolutionary Biology*, **12**, 254.

Zhao, Y.J. & Gong, X. (2012) Genetic structure of the endangered *Leucomeris decora* (Asteraceae) in China inferred from chloroplast and nuclear DNA markers. *Conservation Genetics*, **13**, 271-281.

Note S1. Supplementary information on data analyses

Testing for recombination and linkage disequilibrium of nuclear genes

We used IMgc (Woerner *et al.*, 2007) to generate a reduced, recombination-free matrix, which would be needed for coalescent-based analyses that assume no recombination. We tested for linkage disequilibrium between pairs of loci in both species based on Fisher's method using the program Genepop v. 4.4 (Raymond & Rousset, 1995). The probability was adjusted via Bonferroni correction.

Neutral test of nuclear genes

We used a recently developed maximum frequency of derived mutation (MFDM) method (Li, 2011), to examine the neutrality of variation at each nuclear locus in each species. This method is reliable to distinguish selection from historical demography such as bottlenecks and size expansions, which may produce similar signals to selection. In comparison with other neutrality tests, it showed high power for detecting recent positive selection and was widely used recently (Li *et al.*, 2011; Lopez de Heredia *et al.*, 2014; Ru *et al.*, 2016). For each locus, we used the longest region without recombination in order to decrease the false-positive rate. Moreover, as migration may also cause unbalanced trees, a migration detector (MD) was used to analyze this possibility of the unbalanced tree caused by migration. For each locus in each species, we arbitrarily selected one individual or did not add the individual from another species for the MD analyses to compare the differences of the two methods.

Species tree analysis based on nuclear genes

A species tree was estimated from the joint posterior probability of the nuclear gene trees using software BEAST v. 1.8.2 (Drummond & Rambaut, 2007). We analysed the dataset including all individuals used for nuclear DNA sequencing (see more details in Supporting information). *BEAST requires the priori assignment of individuals to species categories, and because our phylogenetic tree based on chloroplast DNA fragments showed significant intraspecific structure in *L. decora* and its nonmonophyly, we treated each of the two groups of *L. decora* and all *N. insignis* individuals as separate taxonomic units in this analysis. Each locus was treated as a single partition by unlinking gene trees, rates and substitution model and the optimal model of nucleotide substitution was selected by the AIC calculation in jModelTest (Posada, 2008). Uncorrelated lognormal relaxed clock models were used for each locus and a speciation Yule process was specified as tree prior. Multiple independent runs were conducted for 200 million generations sampling every 20,000 generations. The convergence across independent analyses was assessed in TRACER 1.5. We also constructed genealogical relationship at each locus based on haplotypes excluding insertions and deletions using NETWORK v. 5.0 (available at http://www.fluxus-engineering.com).

Coalescent-based analyses

Isolation with migration analyses were implemented in IMa (Hey & Nielsen, 2007). We investigated interspecific gene flow using the two cpDNA fragments and eight nuclear loci after excluding two loci (*A39* and *GA2ox1*), which deviated from the

neutral model with the aid of MFDM neutral test, respectively. The longest nonrecombining regions for each locus obtained from IMgc analyses (Woerner *et al.*, 2007) were used for IMa estimation. The HKY model was chosen for all loci. The two datasets were both used for final demographic parameter estimations. After preliminary runs, convergence upon the stationary distribution was considered to have occurred if independent runs generated similar posterior distributions with the effective sample size (ESS) for each parameters > 100 (Hey & Nielsen 2007). To convert parameter estimations to demographic units, we considered a range of mutation rates and generation time as 5 years. According to the phylogenetic study by Funk et al. (2014), *Ainsliaea* and *Pertya* species were estimated to diverge approximately between 12.5-32.11 Mya. Assuming *Pertya phylicoides* and *Ainsliaea latifolia* diverged within this time range, we first calculated the average divergence at silent sites for individual locus between *Pertya phylicoides* and *Ainsliaea latifolia* (*K*s), and then applied the formula $\mu = K\text{s}/2\text{T}$ to estimate the mutation rate per year at individual nuclear locus. The calculated mutation rates ranged from 1.2×10^{-9} to 3.9×10^{-9}. We note that these estimates are lower than the average values generally reported for nuclear genes (e.g. $5.1\text{-}7.1 \times 10^{-9}$s/s/y) (Graur & Li, 2000), but consistent with notion that woody taxa have slower rates of molecular evolution and for example, the estimation for *GAPDH* ($0.89\text{-}2.23 \times 10^{-9}$) and *AroB* (mean = 1.48×10^{-9}), is close to other woody species like the valley oak ($0.16\text{-}2.3 \times 10^{-9}$) (Gugger *et al.*, 2013) and other conserved ortholog set genes (e.g. *At103*: 1.62×10^{-9} and *Eif3E*: 1.38×10^{-9}) (Duminil *et al.*, 2015), respectively. For cpDNA fragments, the minimum mutation rate (4.87×10^{-10}) investigated by Wolfe *et al.* (1987) and Richardson *et al.* (2001) was used considering the low mutation rate of nuclear loci. The resulting geometric average mutation rate over the eight loci was utilized to scale the effective population size and divergence time. Finally, $1.5\text{-}2.0 \times 10^{8}$ interactions were performed and the first 10% were discarded as burn-in.

We used DIYABC v. 2.1.0 for the ABC-based scenario comparisons (Cornuet *et al.*, 2014) based on the sequence data of the 10 nuclear loci. According to the much lower genetic diversity in *N. insignis* than those of *L. decora*, together with the phylogenetic analyses (see results), two possible scenarios for divergence of *L. decora* and *N. insignis* were compared: (1) *L. decora* and *N. insignis* diverged from the common ancestor and *N. insignis* experienced bottlenecks during the glacial period; (2) *N. insignis* was derived from *L. decora* populations and established its current distribution through recent expansion (Supplementary Data Table S4 and Fig. S2). The detailed setting for this analysis was illustrated in Appendix S4. We used five one-sample summary statistics: number of segregating sites, number of pairwise differences, Tajima's D and private segregating sites; and mean of pairwise differences (W), mean of pairwise differences (B) and F_{ST} were selected as two-sample summary statistics to compare observed and simulated data sets. We simulated 10^{6} data for each scenario and gave each scenario a uniform prior probability. After the simulations, we first run pre-evaluation to check the similarity between the simulated and observed data. Then, the posterior probability of the scenarios was computed using a logistic regression approach on the first 1%

simulations and the scenario with the highest posterior probability was selected as the best. Third, the proportion of type I and type II errors were estimated to determine whether the scenario simulated was correctly identified. Subsequently, we estimated the posterior distributions of parameters under the best scenario. The average generation time was set to 5 years according to our filed observations for the two species.

CITED REFERENCES IN NOTE S1

Cornuet, J.-M., Pudlo, P., Veyssier, J., Dehne-Garcia, A., Gautier, M., Leblois, R., Marin, J.-M. & Estoup, A. (2014) DIYABC v2.0: a software to make approximate Bayesian computation inferences about population history using single nucleotide polymorphism, DNA sequence and microsatellite data. *Bioinformatics*, **30**, 1187-1189.

Drummond, A.J. & Rambaut, A. (2007) BEAST: Bayesian evolutionary analysis by sampling trees. *BMC Evolutionary Biology*, **7**, 214.

Duminil, J., Mona, S., Mardulyn, P., Doumenge, C., Walmacq, F., Doucet, J.-L. & Hardy, O.J. (2015) Late Pleistocene molecular dating of past population fragmentation and demographic changes in African rain forest tree species supports the forest refuge hypothesis. *Journal of Biogeography*, **42**, 1443-1454.

Funk, V.A., Sancho, G., Roque, N., Kelloff, C.L., Ventosa-Rodriguez, I., Diazgranados, M., Bonifacino, J.M. & Chan, R. (2014) A phylogeny of the Gochnatieae: Understanding a critically placed tribe in the Compositae. *Taxon*, **63**, 859-882.

Graur, D. & Li, W.H. (2000) *Fundamentals of molecular evolution*. Sinauer Associates, Sunderland, Massachusetts.

Gugger, P.F., Ikegami, M. & Sork, V.L. (2013) Influence of late Quaternary climate change on present patterns of genetic variation in valley oak, *Quercus lobata* Née. *Molecular Ecology*, **22**, 3598-3612.

Hey, J. & Nielsen, R. (2007) Integration within the Felsenstein equation for improved Markov chain Monte Carlo methods in population genetics. *Proceedings of the National Academy of Sciences, USA*, **104**, 2785-2790.

Li, H.P. (2011) A new test for detecting recent positive selection that is free from the confounding impacts of demography. *Molecular Biology and Evolution*, **28**, 365-375.

Li, Z.H., Zou, J.B., Mao, K.S., Lin, K., Li, H.P., Liu, J.Q., Kallman, T. & Lascoux, M. (2011) Population genetic evidence for complex evolutionary histories of four high altitude Juniper species in the Qinghai-Tibetan Plateau. *Evolution*, **66**, 831-845.

Lopez de Heredia, U., Lopez, R., Collada, C., Emerson, B.C. & Gil, L. (2014) Signatures of volcanism and aridity in the evolution of an insular pine (*Pinus canariensis* Chr. Sm. Ex DC in Buch). *Heredity*, **113**, 240-249.

Posada, D. (2008) jModelTest: phylogenetic model averaging. *Molecular Biology and Evolution*, **25**, 1253-1256.

Raymond, M. & Rousset, F. (1995) An exact test for population differentiation. *Evolution*, **49**, 1280-1283.

Richardson, J.E., Pennington, R.T., Pennington, T.D. & Hollingsworth, P.M. (2001) Rapid diversification of a species-rich genus of neotropical rain forest trees. *Science*, **293**, 2242-2245.

Ru, D.F., Mao, K.S., Zhang, L., Wang, X.J., Lu, Z.Q. & Sun, Y.S. (2016) Genomic evidence for polyphyletic origins and interlineage gene flow within complex taxa: a case study of *Picea brachytyla* in the Qinghai-Tibet Plateau. *Molecular Ecology*, **25**, 2373-2386.

Woerner, A.E., Cox, M.P. & Hammer, M.F. (2007) Recombination-filtered genomic datasets by information maximization. *Bioinformatics*, **23**, 1851-1853.

Wolfe, K.H., Li, W.H. & Sharp, P.M. (1987) Rates of nucleotide substitution vary greatly among plant mitochondrial, chloroplast, and nuclear DNAs. *Proceedings of the National Academy of Sciences, USA*, **84**, 9054-9058.
